# Open questions: What has genetics told us about autism spectrum disorders?

**DOI:** 10.1186/1741-7007-12-45

**Published:** 2014-06-05

**Authors:** Martin Raff

**Affiliations:** 1 MRC Laboratory for Molecular and Cell Biology, University College London, Gower Street, London WC1E 6BT, UK

## Abstract

Some of the most interesting questions in biology today, in my view, derive from the real advances in neuropsychiatry that have come largely from human genetics. Research in autism spectrum disorders (ASDs) has been leading the way, mainly because it has become especially well funded and has recently attracted many outstanding scientists. (I must make it clear that I am an outsider in this field, as I have never worked on any neuropsychiatric disorder).

## Autism spectrum disorder genetics

More than 25 mutations that have a large effect on risk have now been strongly implicated in ASDs. Some are inherited, whereas others are *de novo*[1-5]. All of them are rare: no one of the affected genes appears to be involved in more than 1% of ASDs. Interestingly, so far, none of them has been found to be specific for ASDs, in that most have also been implicated in other neuropsychiatric disorders, including, for example, intellectual disability, schizophrenia and epilepsy. Even in the same family, the same large-effect mutations can contribute to different neuropsychiatric disorders. There thus seems to be a deep genetic connection between these disorders that is not understood. One possibility is that they all reflect synaptic dysfunction: there is increasing evidence, for example, that mutations that affect genes that encode proteins that work only or primarily at synapses can greatly increase the risk of ASDs and of other neuropsychiatric disorders [5].

From analyses of copy number variants (CNVs) and exome sequencing, it is now estimated that mutations in hundreds of genes can substantially increase the risk of developing ASD, the predisposing mutant genes varying from one individual to another [1-5]. Thus, ASDs are genetically heterogeneous, and their well known clinical heterogeneity is presumably at least in part a reflection of this genetic heterogeneity. There is also evidence that more than one rare, large-effect mutation can contribute to ASD in a single individual. So although ASDs are common (approximately 1% prevalence), the genetic underpinning in each individual is rare, and may even be unique, much as an individual cancer is genetically unique. All of this is remarkable progress, given that, until recently, very little was known about ASD genetics.

## Reversibility in monogenetic animal models

Once a large-effect mutation is identified as a risk factor for a neuropsychiatric disorder, one can make a genetic animal model of it, in which the underlying neurobiological basis of the disorder can be studied. Such approaches have already yielded important insights into the nature of ASDs. The studies on mouse models of the monogenetic, or syndromic, forms of these disorders - such as Rett and fragile X syndromes - have been particularly informative. These single-gene conditions are neurologically complex, and the core features of autism occur in about half of the affected individuals. The most dramatic and surprising finding has been that both conditions can be largely reversed in adult mice carrying the mutations: if the brain problem is corrected either genetically (in Rett, where the mutation is in *MeCP2*) [6] or with drugs that correct an abnormal downstream signaling pathway (in fragile X, where the mutant gene is *FMR1*) [7], most of the behavioral and physiological abnormalities go away. This was totally unexpected, as these are developmental brain disorders: but these remarkable results demonstrate that the serious neurological defects seen in these mutant mice reflect reversible functional defects in the adult brain. This is potentially very good news for the future of drug therapies for humans with ASDs (and probably for some other neuropsychiatric disorders as well), although it is still uncertain whether ASDs in humans are similarly reversible in adults.

If neuropsychiatric disorders are primarily the result of synaptic dysfunction, however, reversibility should not be so surprising: synaptic plasticity is, after all, presumably the basis for all of normal brain function that depends on memory, and it persists well into adulthood, which is why even as we age we can still learn and remember - at least some things. Moreover, some human neuropsychiatric disorders spontaneously show dramatic, if temporary, reversibility. Bipolar disorder is the clearest example: in this condition, individuals can switch from extreme mania to deep depression and then to apparent normality, and can do this repeatedly. It is clear that the problem in bipolar disorder is an abnormality in mood control, but we don’t know much about the normal mood control system, let alone how it goes wrong in bipolar disorder. The identification of large-effect mutations that greatly increase the risk of this disorder, however, could lead to useful animal models, which might then shed light on both the underlying brain abnormality in bipolar individuals and the normal mood control system.

## If there is a synaptic problem in ASDs, what is it?

If synaptic dysfunction is at the heart of ASDs, what exactly is the synaptic problem? Given the clinical and genetic heterogeneity of ASDs, there is unlikely to be one answer. There is evidence that there are defects in the ratio of excitatory to inhibitory synaptic transmission in some mouse ASD models, but it seems that the ratio can be abnormally increased or decreased, and the abnormality can vary from one brain region to another, even in the same mouse model [8].

Even if a consistent synaptic defect were found, it would be a major challenge to relate it to a particular behavioral phenotype. And how would you know whether the defect is a primary result of the genetic defect or a secondary effect of the brain’s attempt to compensate? After all, the nervous system is a specialist in compensatory adjustments.

## Are abnormal genes the whole story?

Although genetic abnormalities are likely to play an important part in most ASDs, they are unlikely to be the only contributor. If one identical twin develops an ASD, there is a 70 to 90% chance that the other will too - but it’s not 100%. Why not? The answer isn’t known, but the acquisition of somatic mutations in the developing brain could, in principle, be part of the answer.

Could environmental influences play a part? Although there is no compelling evidence that adverse postnatal environmental factors contribute to the risk of ASDs, there is strong evidence that stressful conditions *in utero* can do so: maternal infection early in pregnancy, for example, can greatly increase the risk of ASDs (and schizophrenia) [9]. It remains a mystery how such stresses *in utero* interact with predisposition genes in the fetus.

## Why the ASD ‘core’?

ASD individuals have two defining characteristics: impaired social communication and interactions; and restricted and obsessive interests and behaviors. Together, these constitute the autistic ‘core’. In addition, most individuals on the autistic spectrum have a number of other neurological and psychiatric problems, which can include intellectual disability, language impairment, sensory and motor abnormalities, and so on.

A central mystery in ASDs is why mutations in so many genes result in the core features of ASD. It is not obvious why these features should occur together. What connects them? There are various possible explanations. One possibility is that the primary problem is the strong drive for sameness and predictability (which is an important part of the restricted interests and obsessive behaviors phenotype), and this leads to a secondary social withdrawal; after all, people are the least predictable objects in a developing child’s environment. Another possibility is that the primary problem is an electrically 'noisy' brain, which leads to a secondary social withdrawal and restricted interests - to keep the noise level to a minimum.

Although many mysteries remain, considering the little that was known up to a decade or so ago, there has been staggering recent progress, which is bringing the neurobiology of ASDs and neuropsychiatric disorders out of the darkness into the light. Much of what has been learned so far is encouraging, and, as more high-risk genes are discovered, progress can only accelerate.

## References

[B1] Pinto D Delaby E Merico D Barbosa M Merikangas A Klei L Thiruvahindrapuram B Xu X Ziman R Wang Z Vorstman JA Thompson A Regan R Pilorge M Pellecchia G Pagnamenta AT Oliveira B Marshall CR Magalhaes TR Lowe JK Howe JL Griswold AJ Gilbert J Duketis E Dombroski BA De Jonge MV Cuccaro M Crawford EL Correia CT Conroy J Convergence of genes and cellular pathways dysregulated in autism spectrum disorders Am J Hum Gene 2014 12 677 694 10.1016/j.ajhg.2014.03.018 PMC4067558 24768552

[B2] Ronemus M Iossifov I Levy D Wigler M The role of *de novo* mutations in the genetics of autism spectrum disorders Nat Rev Genet 2014 12 133 141 10.1038/nrg3585 24430941

[B3] Krumm N O’Roak BJ Shendure J Eichler EE A *de novo* convergence of autism genetics and molecular neuroscience Trends Neurosci 2014 12 95 105 10.1016/j.tins.2013.11.005 24387789 PMC4077788

[B4] Yu TW Chahrour MH Coulter ME Jiralerspong S Okamura-Ikeda K Ataman B Schmitz-Abe K Harmin DA Adli M Malik AN D'Gama AM Lim ET Sanders SJ Mochida GH Partlow JN Sunu CM Felie JM Rodriguez J Nasir RH Ware J Joseph RM Hill RS Kwan BY Al-Saffar M Mukaddes NM Hashmi A Balkhy S Gascon GG Hisama FM LeClair E Using whole-exome sequencing to identify inherited causes of autism Neuron 2013 12 259 273 10.1016/j.neuron.2012.11.002 23352163 PMC3694430

[B5] Guillaume H Ey E Bourgeron T The genetic landscapes of autism spectrum disorders Annu Rev Genomics Hum Genet 2013 12 191 213 10.1146/annurev-genom-091212-153431 23875794

[B6] Guy J Gan J Selfridge J Cobb S Bird A Reversal of neurological defects in a mouse model of Rett syndrome Science 2007 12 1143 1147 10.1126/science.1138389 17289941 PMC7610836

[B7] Michalon AI Siderov M Ballard TM Ozmen L Spooren W Wettstein JG Jaeschke G Bear MF Lindemann L Chronic pharmacological mGlu5 inhibition corrects fragile X in adult mice Neuron 2012 12 49 56 10.1016/j.neuron.2012.03.009 22500629 PMC8822597

[B8] Etherton M Földy C Sharma M Tabuchi K Liu X Shamloo M Malenka RC Südhof TC Autism-linked neuroligin-3 R451C mutation differentially alters hippocampal and cortical synaptic function Proc Natl Acad Sci U S A 2011 12 13764 13769 10.1073/pnas.1111093108 21808020 PMC3158170

[B9] Patterson PH Maternal infection and immune involvement in autism Trends Mol Med 2011 12 389 394 10.1016/j.molmed.2011.03.001 21482187 PMC3135697

